# A case of scleritis associated rheumatoid arthritis accompanying an intraocular elevated lesion

**DOI:** 10.1186/s12886-018-0797-z

**Published:** 2018-05-30

**Authors:** Takatoshi Kobayashi, Nanae Takai, Rei Tada, Hiromi Shoda, Teruyo Kida, Tsunehiko Ikeda, Takurou Ozaki, Shigeki Makino

**Affiliations:** 1 Department of Ophthalmology, Osaka Medical College, 2-7 Daigaku-machi, Takatsuki City, Osaka 569-8686 Japan; 2 Tada Eye Clinic, Ikeda City, Japan; 3 Department of Internal Medicine, Osaka Medical College, Takatsuki City, Japan

**Keywords:** Scleritis, Uveitis, Rheumatoid arthritis, Granuloma, Intraocular tumor

## Abstract

**Background:**

Scleritis and/or uveitis sometimes accompanies patients who suffer from rheumatoid arthritis. However, few studies have reported scleritis and/or uveitis accompanying a fundus elevated lesion, such as an intraocular tumor. In this study, we report a case of rheumatoid uveitis associated with an intraocular elevated lesion.

**Case presentation:**

A 66-year-old female visited another eye clinic and was diagnosed as bilateral anterior uveitis, and was prescribed steroid eye drops for treatment. She had previously been diagnosed as rheumatoid arthritis at the age of 30 years. Due to vitreous opacity that appeared in her right eye, we increased the instillation of steroid eye drops and the amount of oral prednisolone. Although the inflammation had improved, anterior uveitis relapsed, and an intraocular whitish elevated lesion resembling an intraocular tumor at the superior nasal retina appeared. We speculated this lesion to be a granuloma complicated with rheumatoid arthritis. Thus, we increased the amount of prednisolone administration, and the lesion began to shrink and ultimately fully disappeared.

**Conclusions:**

We strongly believe that our case’s lesion was a subretinal granuloma related with rheumatoid arthritis, as it disappeared by increased corticosteroid treatment. Our findings show that we should consider rheumatoid arthritis in a differential diagnosis of such types of fundus elevated lesions.

## Background

Rheumatoid arthritis is a collagen disease, and is one of the autoimmune disorders characterized by persistent synovitis, systemic inflammation, and autoantibodies [1]. Ophthalmologists sometimes examine patients suffering from rheumatoid arthritis combined with ocular inflammation, such as keratoconjunctivitis sicca, episcleritis, scleritis, or uveitis as an extra-articular disease [1, 2].

It is thought that scleritis is caused by the immune-complex deposition, as it reportedly has been found in tissues from vasculitis in necrotizing scleritis-associated collagen disease, such as rheumatoid arthritis [3]. Thus, immunosuppressive therapies including corticosteroids are the primary therapeutic procedures used to treat such scleritis cases. Most cases of scleritis follow good clinical courses, but some cases are refractory, which cannot be cured despite the administration of immunosuppressants. Moreover, there are some cases that are even difficult to diagnose as scleritis. For example, some studies have reported cases of posterior scleritis which were diagnosed as an intraocular tumor [4]. However, a few studies have reported an elevated-lesion-like intraocular tumor that developed following scleritis and/or uveitis. In this present study, we report a rare case of uveitis-associated rheumatoid arthritis in which an intraocular elevated lesion occurred, although the uveitis had once subsided after steroid therapy.

## Case presentation

The present case involved a 66-year-old female who became aware of decreased vision in her left eye. She had previously visited another eye clinic, and was diagnosed as bilateral anterior scleritis and prescribed steroid eye drops for treatment. Although the inflammation had subsided and the scleral redness had disappeared, vitreous opacity increased in her right eye around August 2014. Thus, she was diagnosed as uveitis and referred to the Department of Ophthalmology at Osaka Medical College in September 2014 for examination.

The patient had previously been diagnosed with rheumatoid arthritis when she was 30 years of age, and she had undergone a 5 mg-per-day administration of prednisolone as a maintenance dose and long-term treatment with immunosuppressants such as tacrolimus and anti-tumor necrosis factor (anti-TNF) drugs such as etanercept by the Department of Internal Medicine at our university hospital. Her past medical history included phacoemulsification and aspiration with bilateral intraocular lens (IOL) implantation surgery in January 2014. It should be noted that there was no significant family history.

**Fig. 1:**
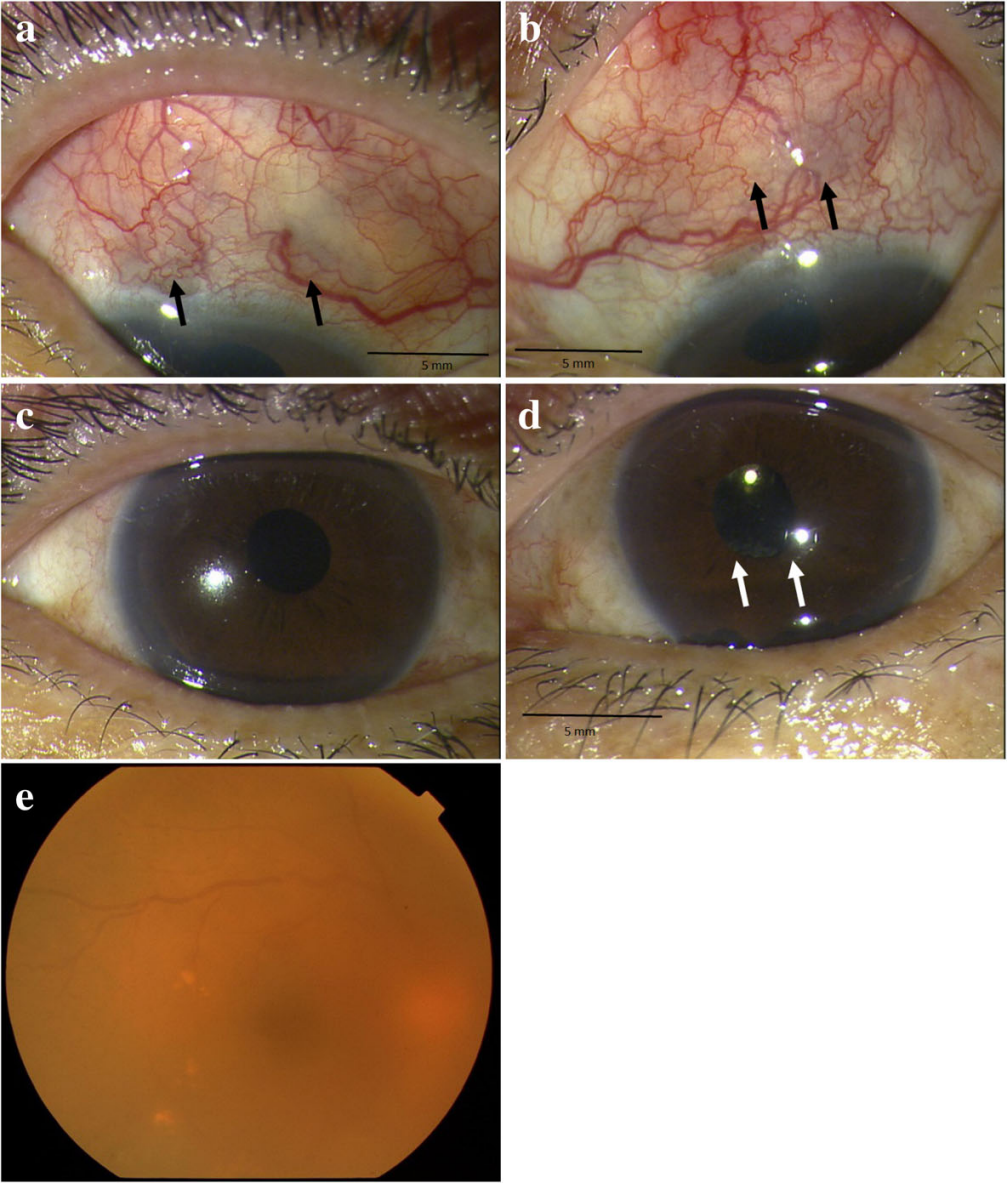
Slit-lamp and fundus photographs obtained at the initial examination of the 66-year-old female patient. **a** Superior sclera in her right eye. **b** Superior sclera in her left eye. **c** Frontal view of her right eye. **d** Frontal view of her left eye. **e** Vitreous opacity in her right eye. The superior sclera in both eyes showed redness and thinning of tissues (arrows). The iris in her left eye was found to be adhered to the implanted IOL (arrows)

Upon initial ocular examination, her visual acuity (VA) was 20/50 × S-0.25D = C-2.00D Ax90° in the right eye and 20/25 × S-0.50D = C-1.25DAx100° in the left eye, and intraocular pressure was 17 mmHg OD and 15 mmHg OS. Slit-lamp examination revealed 1+ cells in both anterior chambers, and fine keratic precipitates on both corneas. The superior sclera in both eyes showed redness and thinning of tissues (Fig. 1a, b). Moreover, the iris in her left eye was found to be adhered to the implanted IOL (Fig. 1c, d). There was no remarkable fundus abnormality except for moderate vitreous opacity in her right eye (Fig. 1e). Blood tests revealed negative results for tuberculosis and syphilis.

**Fig. 2:**
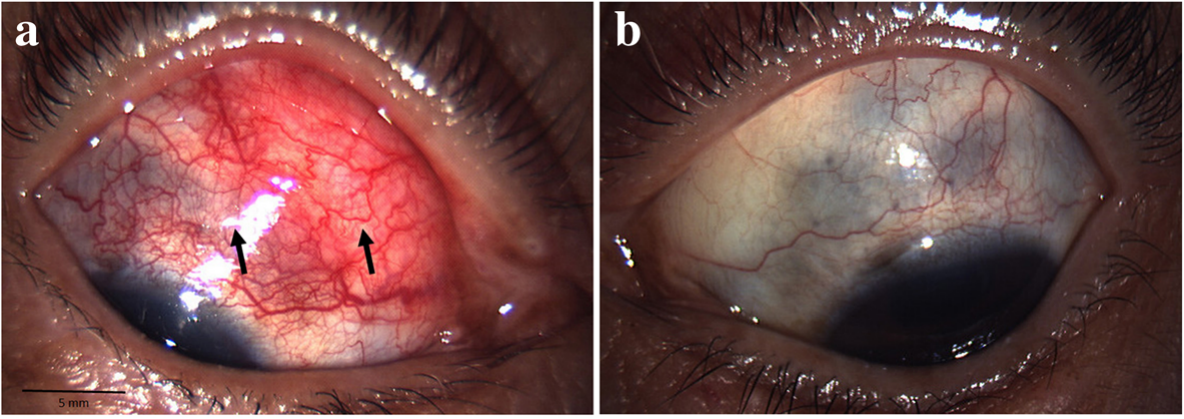
Slit-lamp photographs obtained when scleritis relapse, accompanied with an intraocular elevated lesion. **a** Superior sclera in her right eye, showing redness and a relatively large nodule (arrows). **b** Superior sclera in her left eye, showing the thinning of tissues (arrows)

**Fig. 3:**
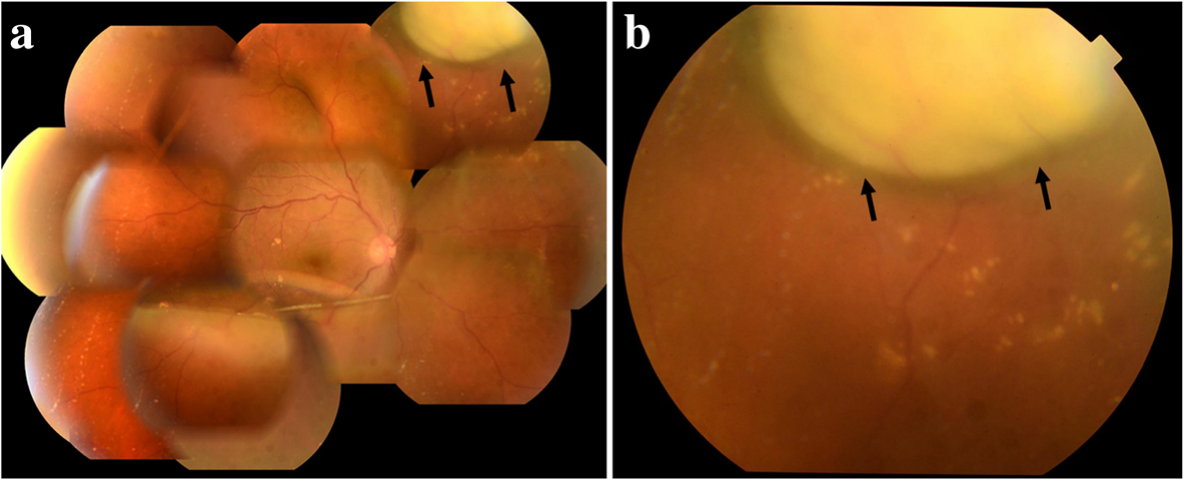
Fundus photographs obtained when scleritis relapsed, accompanied with an intraocular elevated lesion. **a** Intraocular elevated lesion at the superior nasal retina (arrows). **b** Enlarged photograph of the intraocular elevated lesion (arrows)

**Fig. 4:**
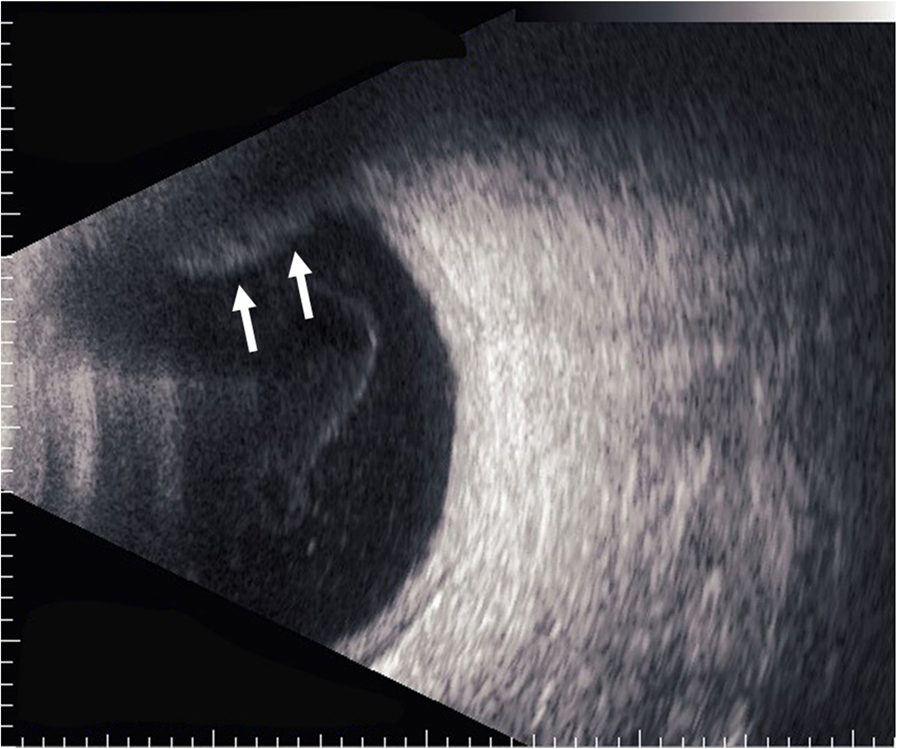
B-scan ultrasonography showed that the sclera was thickened and that the lesion seemed to have high internal reflectivity (arrows)

In regard to the follow-up treatment course, we initially increased the frequency of the instillation of steroid eye drops and added immunosuppressive eye drops. However, those drugs were ineffective, and the vitreous opacity gradually increased. Thus, we increased the amount of oral prednisolone to 20 mg per day in November 2014. Subsequently, the eye redness and vitreous opacity gradually disappeared within approximately 2 weeks, her VA slowly improved, and there was no remarkable fundus abnormality in the right eye at each examination. However, uveitis accompanied with moderate ocular pain relapsed (Fig. 2a, b), and fundoscopic examination revealed an intraocular elevated whitish lesion at the superior nasal retina of her right eye in November 2015 (Fig. 3a, b). We did not observe any restricted motility accompanied with eye movement. B-scan ultrasonography was also performed, and revealed that the sclera was thickened and that the lesion seemed to have high internal reflectivity (Fig. 4). Although the patient underwent a magnetic resonance imaging (MRI) scan for a differential diagnosis, it was difficult to distinguish whether the lesion was a granuloma or a tumor. Ultrasound biomicroscopy (UBM) and high frequency B scan might have been useful to distinguish between a tumor and granuloma, however, those examinations were not available at that time.

**Fig. 5:**
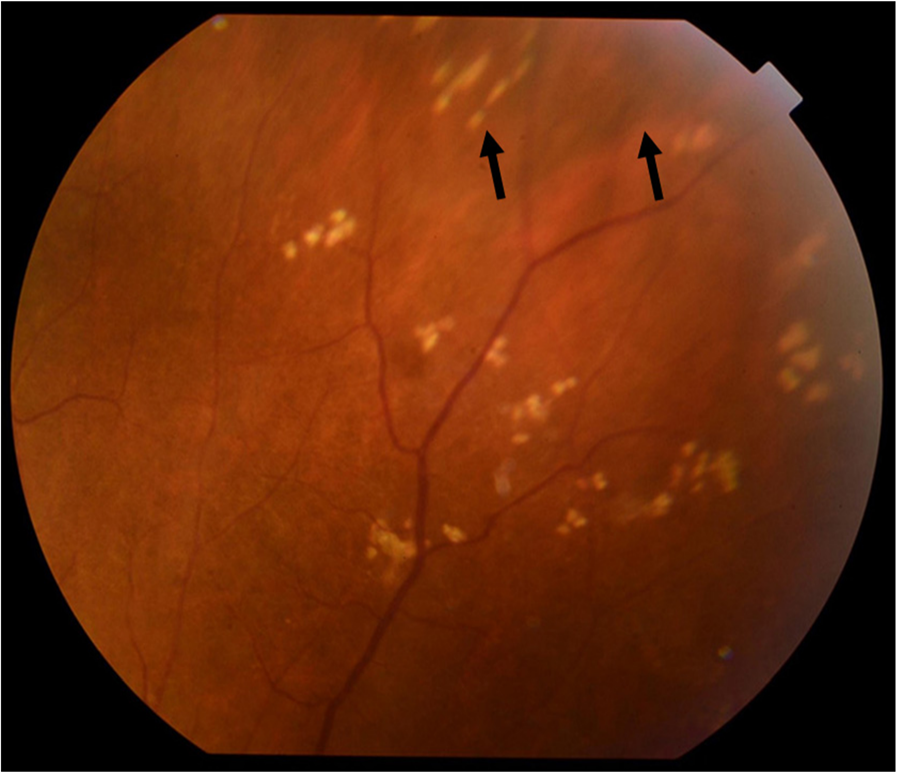
Fundus photograph obtained after disappearance of the intraocular elevated lesion (arrows)

Hence, we recommended to the patient that she should undergo fluorescein and indocyanine angiography examination for differential diagnosis, however, the patient wished to receive treatment without undergoing those examinations. Both the value of C-reactive protein (0.60 mg/dL) and the blood sedimentation rate (32 mm per hour) were increasing. In addition, the value of matrix metalloproteinase-3 (135.0 ng/mL), an indicator of the activity of rheumatoid arthritis, was also increasing. After consultation with her rheumatologist, we increased the administration amount of prednisolone to 30 mg per day. As a result, the lesion began to shrink 1-week after, and fully disappeared 4-weeks after, initiating the increased administration (Fig. 5). Her VA improved to 20/20, and there has been no recurrence of ocular inflammation up to the present time.

## Discussion

Rheumatoid arthritis is known to be a representative disorder accompanying scleritis and/or uveitis. In Japan, rheumatoid arthritis occupies the first position of etiology of scleritis, except idiopathic [5]. Scleritis is categorized as episcleritis, anterior scleritis, or posterior scleritis, depending on location at onset. In addition, anterior scleritis is also categorized as nodular, diffuse, and necrotizing scleritis, depending on the pathogenesis [6]. In cases of rheumatoid arthritis, anterior scleritis is more frequent than posterior scleritis, and inflammation is likely to occur in the superior sclera [7, 8].

In addition, when a case of scleritis and/or uveitis has possibly been caused by systemic disorders such as collagen disease or infectious disease, treatment of those systemic disorders is also essential. Even when the systemic disorders are relatively stable, ocular inflammation may occur, such as in our present case, and the treatment of such patients must be conducted in close cooperation with internal physicians or rheumatologists. In this study, we wish to emphasize that it is most important to distinguish whether or not the uveitis is caused by infectious inflammation. Some studies have reported cases of infectious inflammation, such as tuberculosis or syphilis, caused by nodular infectious uveitis [9, 10]. Biswas et al. reported a case of tuberculous uveitis associated with rheumatoid arthritis that resulted in enucleation of the eye [9]. It is now possible for the life prognosis of the patients who suffer from rheumatoid arthritis to improve thanks to medical advancements, so the opportunities for ophthalmologists to examine such patients who have taken immunosuppressants for a few decades will increase in the future.

When treating scleritis and /or uveitis accompanying such an elevated lesion, it is vital to first distinguish non-infectious scleritis/or uveitis from infectious diseases. In the present case, blood examinations revealed negative results for tuberculosis and syphilis. However, it is sometimes difficult to distinguish between the two, and we usually hesitate to increase the amount of corticosteroid administered in such cases. Liu et al. reported that a patient with a lesion similar to the one in our case improved with a nonsteroidal anti-inflammatory drug (NSAID) [11]. Since it is thought that NSAIDs produce fewer side effects than corticosteroids, it might also be better to try NSAID administration in our present case.

Secondly, it is vital to distinguish such an elevated lesion from an intraocular tumor, e.g., malignant melanoma, metastatic choroidal tumor, and malignant lymphoma, because those may occur with no relation to systemic disorders. However, it is sometimes difficult to distinguish between them. In our present case, we were unable to perform fluorescein and indocyanine angiography when the lesion appeared. Sin et al. reported that ocular pain is a useful symptom for differentiating nodular posterior scleritis from other forms of choroidal masses [12], and our patient also reported ocular pain when the lesion appeared.

And thirdly, it is vital to understand that some types of uveitis may occur with a giant elevated lesion, as Sridharan et al. reported in a 53-year-old female case of posterior scleritis [13]. In that study, the lesion was initially thought to be an amelanotic melanoma. However, after various further examinations, she was diagnosed as posterior scleritis with a lesion mimicking malignant melanoma. She was treated with prednisolone, and the lesion completely regressed.

## Conclusion

In this present case, we strongly believe that the elevated lesion was granuloma caused by recurrence of uveitis. Our findings show that we should consider rheumatoid arthritis in the differential diagnosis of such types of fundus elevated lesions.

## Abbreviations

IOL: Intraocular lens
NSAID: Nonsteroidal anti-inflammatory drug
TNF: Tumor necrosis factor
VA: Visual acuity
